# The silent trial - the bridge between bench-to-bedside clinical AI applications

**DOI:** 10.3389/fdgth.2022.929508

**Published:** 2022-08-16

**Authors:** Jethro C. C. Kwong, Lauren Erdman, Adree Khondker, Marta Skreta, Anna Goldenberg, Melissa D. McCradden, Armando J. Lorenzo, Mandy Rickard

**Affiliations:** ^1^ Division of Urology, Department of Surgery, University of Toronto, Toronto, ON, Canada; ^2^ Temerty Centre for AI Research and Education in Medicine, University of Toronto, Toronto, ON, Canada; ^3^ Centre for Computational Medicine, The Hospital for Sick Children, Toronto, ON, Canada; ^4^ Department of Computer Science, University of Toronto, Toronto, Ontario, Canada; ^5^ Division of Urology, Department of Surgery, The Hospital for Sick Children, Toronto, ON, Canada; ^6^ Department of Bioethics, The Hospital for Sick Children, Toronto, ON, Canada; ^7^ Division of Clinical and Public Health, Dalla Lana School of Public Health, University of Toronto, Toronto, ON, Canada; ^8^ Genetics & Genome Biology, Peter Gilgan Centre for Research and Learning, Toronto, ON, Canada

**Keywords:** dataset drift, bias, feasibility, stakeholder attitudes, artificial intelligence

## Abstract

As more artificial intelligence (AI) applications are integrated into healthcare, there is an urgent need for standardization and quality-control measures to ensure a safe and successful transition of these novel tools into clinical practice. We describe the role of the silent trial, which evaluates an AI model on prospective patients in real-time, while the end-users (i.e., clinicians) are blinded to predictions such that they do not influence clinical decision-making. We present our experience in evaluating a previously developed AI model to predict obstructive hydronephrosis in infants using the silent trial. Although the initial model performed poorly on the silent trial dataset (AUC 0.90 to 0.50), the model was refined by exploring issues related to dataset drift, bias, feasibility, and stakeholder attitudes. Specifically, we found a shift in distribution of age, laterality of obstructed kidneys, and change in imaging format. After correction of these issues, model performance improved and remained robust across two independent silent trial datasets (AUC 0.85–0.91). Furthermore, a gap in patient knowledge on how the AI model would be used to augment their care was identified. These concerns helped inform the patient-centered design for the user-interface of the final AI model. Overall, the silent trial serves as an essential bridge between initial model development and clinical trials assessment to evaluate the safety, reliability, and feasibility of the AI model in a minimal risk environment. Future clinical AI applications should make efforts to incorporate this important step prior to embarking on a full-scale clinical trial.

## Introduction

While artificial intelligence (AI) has gained much attention in healthcare, there is a pressing need for standardization and quality-control measures to ensure a safe and successful implementation into clinical practice. Premature deployment of machine learning (ML) models without rigorous external validation and governance can lead to discrepancies between reported and real-world performance, which may ultimately lead to patient harm. A recent example of this is the widely adopted Epic Sepsis Model that was found to have poor discrimination and calibration in predicting the onset of sepsis on external validation (1).

To mitigate these risks, several AI implementation pathways have been described (2, 3). We have previously outlined a 3-stage roadmap for the evaluation and validation of AI models into clinical care (4, 5), which has been implemented at scale at our institution. These phases include (1) exploratory model development, (2) a silent trial, and (3) prospective clinical evaluation. Several guidelines address the first and third phases to help standardize reporting, enhance reproducibility, and reliability of AI studies in healthcare (6–9). However, there has been limited discussion of the role of the silent trial, which evaluates the proposed model on patients in real-time, while the end-users (i.e., clinicians) are blinded to predictions such that they do not influence clinical decision-making. As shown in Table 1, this phase is essential to establishing feasibility and safety of AI models prior to proceeding with clinical evaluation where the model influences patient care.

**Table 1:** Major themes to explore during the silent trial before transitioning to the clinical trial phase. Each theme is associated with a suggested list of questions that should be considered.

Themes	Key questions
**Dataset drift**: Are there any changes between the training dataset and patients evaluated in the silent trial?^a^	1. Are there any changes as to how data are defined and collected? 2. Are there any changes to patient demographics, clinical settings, or unexpected events (i.e.: COVID-19) that would impact the patient population in which the model is applied? 3. Are there any changes in clinical practice such as indication, standard of care, or patient preference, that would influence the data being collected?
**Bias**: Was the model trained on a generalizable dataset to ensure fairness to all patients regardless of gender, race, etc.?	1. Which subset of patients benefit from the model? 2. Which subset of patients are harmed by the model?
**Feasibility:** Can the AI intervention be easily integrated within the existing clinical workflow?	1. How much time does it take for the end-user (i.e.: clinician) to input the necessary variables to generate a prediction? 2. How is the clinical workflow or duration of a clinic visit impacted with the use of the AI intervention? Importantly, does it slow down clinical workflow without a clear benefit? 3. Is the user interface simple enough to be used at point of care with minimal or no training? 4. Are the model predictions easy to understand? Are the model explanations easy to interpret? 5. How much computing resources or infrastructure are required to maintain the AI model at scale?
**Stakeholder attitudes**: Are there any concerns with respect to the use of AI to augment patient care?	1. Does the AI intervention facilitate patient counseling, decision-making, or treatment planning? 2. Are patients comfortable with the use of AI interventions to support their care? 3. What are the patient’s priorities or goals of care regarding their condition and are they addressed by the AI intervention?

^a^ Based on Finlayson et al. (13).

The purpose of this article is to highlight the lessons learned from our experience in validating a previously developed model within the context of the silent trial. Here, we present the development of a classification model to predict obstruction in hydronephrotic kidneys of infants using ultrasound images. The current standard of care for infants with hydronephrosis, defined as swelling of one or both kidneys due to inadequate urinary drainage, involves serial ultrasounds typically every 3–6 months for several years. Patients may also undergo more invasive testing such as a diuretic renogram. While these investigations may provide useful information, the trade-off includes exposing patients to radioisotope and ionizing radiation as well as painful procedures such as venous canulation and urethral catheterization (10). Therefore, our aim was to develop an AI model that could reliably distinguish between self-resolving hydronephrosis vs. those that would ultimately require operative management based on initial kidney ultrasound images, thereby potentially reducing the number of invasive tests and expediting surgical interventions when necessary.

Using our model as a case study, we illustrate how issues related to dataset drift, bias, feasibility, and stakeholder attitudes were identified and addressed. This article is intended for clinicians and ML engineers wishing to gain a deeper understanding of the rationale behind the silent trial and provide insights as to how this phase serves as a bridge between initial model development and clinical trials assessment.

## Materials and methods

### Exploratory model development

We have previously developed a deep learning classification model to predict obstructive hydronephrosis in infants using still images from kidney ultrasound (11). Using sagittal and transverse images as inputs, the model would determine the probability of obstructive hydronephrosis and highlight areas of importance on the ultrasound images *via* GradCAM heatmaps (12). Obstructive hydronephrosis was defined by whether a patient ultimately required operative intervention to relieve the obstruction based on chart review. This tool was intended to be used at point-of-care to support clinical decision-making and patient counseling.

### Silent trial

**Figure 1:**
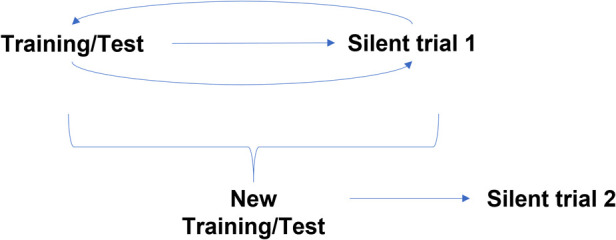
Silent trial workflow for model development. Initially, the model was trained and tested on a random 20% split of the initial dataset. Following successful generalization in this random split, the model was evaluated on new patients using prospectively collected data, Silent Trial 1. From this dataset, we identified any weaknesses in our model preventing it from generalizing successfully and adapted our initial model to overcome these limitations. Once the model generalized in this new set, the model was re-trained on both the initial and Silent Trial 1 datasets. This updated model was then tested on another prospectively collected data set, Silent Trial 2.

Following in silico/algorithmic validation, the AI model was prospectively validated in the silent trial from August to December 2020. During this period, the clinical team assessed and managed patients as per current standard of care. Concurrently, a separate research team recorded model predictions based on ultrasound images obtained at the time of the clinic visit. Additional patient demographics and the clinical decision to proceed with surgery were later collected. The clinical team was blinded to model predictions to avoid influencing clinical decision-making. This “Silent Trial 1” data was used to assess generalization of our initial model in a prospectively collected dataset (Figure 1). The results from Silent Trial 1 were then used to inform the refinement of the original model and data preprocessing steps. Once generalization was achieved on the Silent Trial 1 dataset, the original and Silent Trial 1 datasets were combined for model re-training. This updated model was then evaluated on another prospectively collected dataset, “Silent Trial 2”. Model performance was characterized by area under the receiver-operating characteristic curve (AUROC) and area under the precision-recall curve (AUPRC), along with sensitivity and specificity found at a threshold set in the validation set targeting 90% sensitivity, based on consensus among the clinical expert group. The target of 90% sensitivity was chosen as false negatives would be particularly detrimental. Moreover, assessing model performance with a set threshold allowed us to test our model in a more real-world scenario of decision-making at a specific cut-off, rather than merely noting the separation of obstructed vs. non-obstructed cases.

Given that AI in healthcare is still in its infancy, patient and family attitudes toward AI integration in their urologic care are not well understood. Therefore, it was essential to characterize patient perceptions about these tools to ensure that they were aligned with patient values and their role as a decision-support tool was clearly defined. To explore how patients and families would respond to the introduction of an AI tool into their care, we probed their initial thoughts and values through a standard post-visit follow-up questionnaire (**Supplementary Table S1**). This survey also sought to understand other patient priorities, such as the need for invasive testing, hospital visits, risks of infections, and renal impairment, however these were not the focus of this paper. Similarly, provider attitudes on the value of this AI intervention were assessed through clinical team meetings. We worked with multiple stakeholders in designing the user interface of our AI application. Feasibility was assessed by measuring the average time from starting the AI application to obtaining the probability of obstructive hydronephrosis based on user-uploaded ultrasound images.

## Results

The initial training set contained 1,643 kidneys (1,456 non-obstructed/187 obstructed) from 294 patients (240 non-obstructed/54 obstructed) (Table 2). From a random test set of 20% drawn from the initial training set, the model achieved an AUROC of 90%, AUPRC of 58%, sensitivity of 92%, and specificity of 69% (Table 3, row 1). This model was then evaluated on the Silent Trial 1 dataset, which included 523 kidneys (387 non-obstructed/136 obstructed). This revealed a significant drop in performance with an AUROC of 50%, AUPRC of 26%, sensitivity of 100%, and specificity of 0% (Table 3, row 2). The following sections highlight how the silent trial enabled us to improve model performance and clinical utility by systematically examining the model with respect to dataset drift, bias, feasibility, and stakeholder attitudes.

**Table 2:** Baseline characteristics of each dataset.

	Non-obstructed			Obstructed			Total		
Variable	Training	Silent Trial 1	Silent Trial 2	Training	Silent Trial 1	Silent Trial 2	Training	Silent Trial 1	Silent Trial 2
Sex									
Male	981	326	530	138	104	69	1,119	430	599
Female	247	61	106	42	32	6	289	93	112
Age groups									
<2 years	1,025	359	561	171	128	71	1,196	487	632
2–5 years	143	28	72	9	6	0	152	34	72
>5 years	60	0	3	0	1	3	60	1	6
Ultrasound number									
1	403	127	214	69	46	28	472	173	242
2	316	110	184	50	39	24	366	149	208
3	248	74	130	34	24	11	282	98	141
4	161	39	63	19	12	8	180	51	71
5	112	16	32	8	5	2	120	21	34
6	84	11	10	3	3	1	87	14	11
7	63	6	2	4	3	1	67	9	3
8	37	3	1	0	2	0	37	5	1
9	18	1	0	0	1	0	18	2	0
10	13	0	0	0	1	0	13	1	0
11	1	0	0	0	0	0	1	0	0
Ultrasound Machine									
Philips	891	88	155	101	33	23	992	121	178
Samsung	34	59	125	2	21	17	36	78	125
Toshiba	448	229	347	69	48	32	517	277	379
GE	37	1	0	8	9	1	45	10	1
Acuson	23	0	0	2	0	0	25	0	0
ATL	17	0	0	5	0	0	22	0	0
Siemens	4	0	0	0	0	0	4	0	0
Outside	0	10	9	0	25	2	0	35	11
APD Group									
<6 mm	113	157	284	6	1	0	119	158	284
6–9 mm	119	92	150	6	10	5	125	102	155
9–14 mm	190	69	131	29	34	7	219	103	138
>14 mm	187	64	70	139	90	62	326	154	132
Not measured	847	5	1	7	1	1	854	6	2
Kidney view side									
Right	737	192	222	68	57	14	805	249	236
Left	719	195	414	119	79	61	838	274	475
Hydronephrosis side									
Right	673	126	83	56	52	13	729	178	96
Left	635	143	275	106	61	61	741	204	336
Bilateral	148	118	278	25	23	1	173	141	279
Overall observations	1,456	387	636	187	136	75	1,643	523	711
Overall unique patients	240	105	174	54	45	28	294	150	202

APD, anterior-posterior diameter.

**Table 3:** Iterative model performance.

Row	Train	Test	Model	AUROC	AUPRC	Sensitivity	Specificity
1	Original set	Random 20% from original set	Image only	0.90 (0.85, 0.95)	0.58 (0.39, 0.74)	0.92 (0.81, 1.0)	0.69 (0.63, 0.74)
2	Original set	Silent trial 1	Image only	0.50 (0.50, 0.50)	0.26 (0.21, 0.32)	1.00 (1.00, 1.00)	0.0 (0.0, 0.0)
3	Original set	Silent trial 1	Age and side covariates	0.51 (0.506, 0.52)	0.26 (0.22, 0.32)	1.00 (1.00, 1.00)	0.0 (0.0, 0.0)
4	Original set	Silent trial 1	Age-ablated	0.57 (0.55, 0.59)	0.28 (0.24, 0.35)	1.00 (1.00, 1.00)	0.005 (0.0, 0.01)
5	Original set	Silent trial 1	Side-ablated	0.54 (0.52, 0.55)	0.27 (0.22, 0.34)	1.00 (1.00, 1.00)	0.005 (0.0, 0.01)
6	Original set	Silent trial 1	Revised data prep, with covariates	0.85 (0.81, 0.88)	0.67 (0.58, 0.75)	0.98 (0.95, 1.00)	0.32 (0.27, 0.36)
7	Original set	Silent trial 1	Revised data prep, image only	0.84 (0.80, 0.88)	0.65 (0.57, 0.74)	0.99 (0.96, 1.00)	0.26 (0.22, 0.31)
8	Original set + silent trial 1	Silent trial 2	Revised data prep, with covariates	0.91 (0.88, 0.94)	0.52 (0.41, 0.64)	0.97 (0.93, 1.00)	0.54 (0.50, 0.57)
9	Original set + silent trial 1	Silent trial 2	Revised data prep, image only	0.92 (0.88, 0.95)	0.52 (0.41, 0.64)	0.99 (0.95, 1.00)	0.52 (0.48, 0.56)

Values reflect performance of data in the Test column. Model formulation described in the Model column, indicating iterative experiments performed to rescue Silent trial performance. Sensitivity and specificity thresholds set in validation set targeting 90% sensitivity.

### Dataset drift

**Figure 2:**
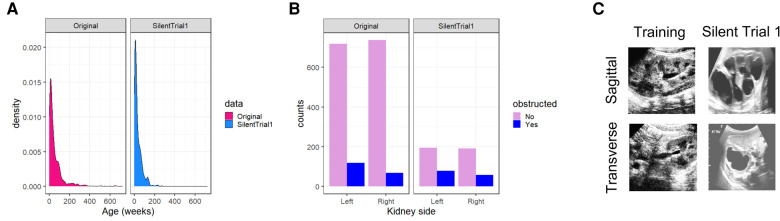
Dataset drift between our original training set and Silent Trial 1. (**A**) The shift in age to younger individuals in the Silent Trial 1 dataset. (**B**) The shift between left and right-sided kidneys in which a larger proportion of right-sided obstructed kidneys were found relative to the left in the Silent Trial 1 set. (**C)** The qualitative shift in images despite the same cropping and normalization procedures for both datasets.

Through multidisciplinary discussions, we hypothesized several reasons for this change in performance including a shift in (1) age distribution, (2) distribution of laterality of obstructed kidneys, and (3) a change in processing of the input images (Figure 2). Indeed, patients included in the Silent Trial 1 dataset were younger (35 ± 39 vs. 61 ± 92 weeks, *p* < 0.01, Figure 2A), had predominantly right-sided obstructed kidneys (42 vs. 36%, *p* < 0.01, Figure 2B), and were visually different even following the same preprocessing steps (Figure 2C). Therefore, we postulated that these differences may explain the precipitous drop in model performance.

**Figure 3:**
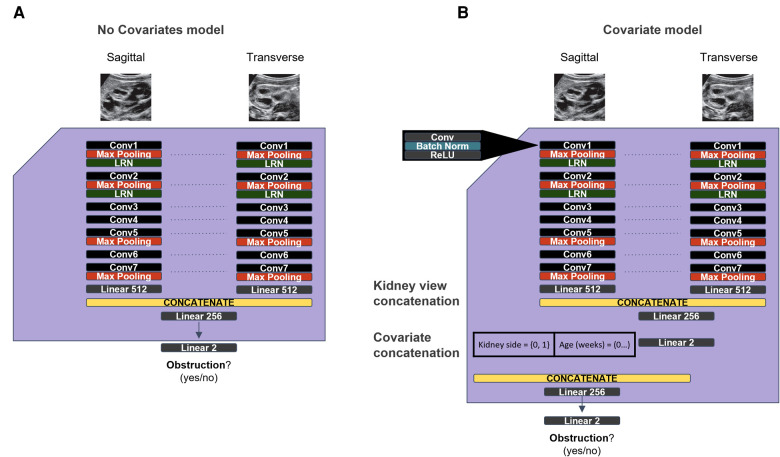
Original and updated models used to overcome dataset drift. (**A**) The original model used from the initial dataset. (**B**) Updated model with covariates for age and kidney laterality, with the goal of overcoming the generalization failure observed on the Silent Trial 1 dataset.

To overcome the limitations of the original model in the Silent Trial 1 dataset, we adapted the original model to incorporate kidney laterality and patient age as covariates to adjust for the dataset drift (Figure 3). With this approach, we found a minor improvement in AUROC, although other performance metrics remained unchanged (Table 3, row 3). We then ablated each covariate by setting either all age or laterality values to zero to evaluate the degree to which each covariate impacted the model's performance, with the hypothesis that one may be more impactful than the other. This procedure resulted in a small but significant increase in model performance for each ablation (Table 3, rows 4–5).

We next turned to image preprocessing and found that the original dataset included processed jpeg files, whereas the Silent Trial 1 dataset included either unprocessed or processed png files. We first experimented with merely passing these images through the same preprocessing steps and reading them into the model. However, this had clearly not addressed the shift in image formatting. Therefore, we experimented with adding the additional step of saving our newly processed data as jpegs files and re-reading them into the model in the same format. This approach led to a tremendous boost in performance on the Silent Trial 1 dataset, with an AUROC of 85% for the model with covariates and 84% for the image-only model (Table 3, rows 6–7).

After addressing these dataset drift issues, we evaluated these updated models on a third dataset, Silent Trial 2, to confirm the generalizability of this approach and assess if covariates should continue to be included. We found that these models do indeed perform well on the Silent Trial 2 dataset, with an AUROC of 91% for the model with covariates and 92% for the image-only model (Table 3, rows 8–9).

### Bias

Next, we conducted a bias assessment of our model to ensure there were no substantial differences in performance when stratified by clinically relevant subgroups including sex, side of hydronephrosis, ultrasound machine, and patient postal code (Table 4). We find in all cases >90% sensitivity for each subgroup, therefore supporting the overall safety of our model. Specificity is far more variable, however in all cases, we find it >50%, therefore every group would benefit from safe and effective streamlined care with this model.

**Table 4:** Bias assessment of our final AI model.

Variable	AUROC	AURPC	Sensitivity	Specificity
Sex				
Male	0.91 (0.87, 0.94)	0.52 (0.42, 0.65)	0.97 (0.93, 1.00)	0.53 (0.49, 0.57)
Female	0.96 (0.91, 1.00)	0.38 (0.12, 0.80)	1.00 (1.00, 1.00)	0.59 (0.50, 0.68)
Side of hydronephrosis				
Left	0.88 (0.84, 0.93)	0.57 (0.45, 0.72)	0.97 (0.92, 1.00)	0.48 (0.43, 0.53)
Right	0.96 (0.91, 0.99)	0.61 (0.39, 0.86)	1.00 (1.00, 1.00)	0.60 (0.50, 0.71)
Both	0.98 (0.96, 0.99)	0.08 (0.05, 0.30)	1.00 (1.00, 1.00)	0.58 (0.52, 0.63)
Ultrasound machine				
Philips	0.89 (0.83, 0.95)	0.50 (0.31, 0.71)	0.96 (0.84, 1.00)	0.53 (0.46, 0.62)
Samsung	0.92 (0.86, 0.96)	0.50 (0.30, 0.71)	1.00 (1.00, 1.00)	0.58 (0.50, 0.66)
Toshiba	0.93 (0.86, 0.97)	0.53 (0.39, 0.72)	0.97 (0.90, 1.00)	0.53 (0.48, 0.58)
Postal code				
K	1.00 (1.00, 1.00)	0.86 (0.67, 0.91)	1.00 (1.00, 1.00)	0.50 (0.24, 0.82)
L	0.90 (0.85, 0.95)	0.49 (0.37, 0.65)	0.95 (0.89, 1.00)	0.58 (0.52, 0.63)
M	0.91 (0.86, 0.97)	0.57 (0.35, 0.76)	1.00 (1.00, 1.00)	0.50 (0.45, 0.56)
N	NA	NA	NA	0.75 (0.25, 1.00)
P	1.00 (1.00, 1.00)	0.86 (0.00, 0.91)	1.00 (1.00, 1.00)	0.57 (0.24, 0.89)

Performance of our model was stratified by sex, side of hydronephrosis, ultrasound machine, and postal code in our Silent Trial 2 set.

### Feasibility

**Figure 4:**
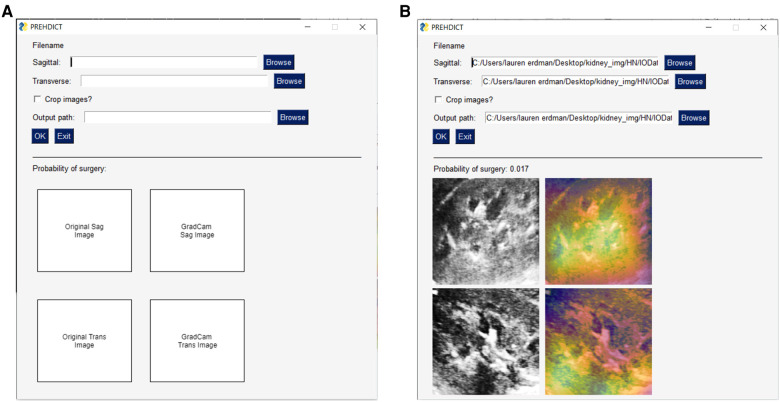
User-interface for image-only model. A basic user-interface was developed to allow clinicians and researchers who are not computer scientists to test the model. (**A**) The user-interface with no data input, in which a user can specify a sagittal and transverse ultrasound image file of the kidney, along with an option for the program to further crop the image and where to save the output. The lower-half of the interface is blank at this point, as it will display the uploaded images. (**B**) This view now shows the user-interface once the model has run. This displays the probability of surgery, the original input images following the preprocessing procedure, and a gradient-based class activation maps to the image to indicate which part of the image is most important for the prediction.

To ensure that the AI intervention was appropriate for routine clinical use, we considered whether the application was simple-to-use and minimally disruptive to the existing workflow. Feasibility was assessed by diverse stakeholders including clinicians, nurse practitioners, trainees, computer scientists, web developers, and patient representatives. The user interface for the AI application was developed using an iterative process involving all stakeholders to simplify instructions, improve clinical utility, and protect patient confidentiality (Figure 4). The average time to generate a model prediction from start-to-finish without prior training was less than one minute. Model output is saved locally within the computer that the program runs on and is analyzed without sending any data over the internet, therefore data and patient-specific findings remain confidential and secure.

### Stakeholder attitudes

Understanding the views and perspectives of patients and providers were essential to ethical integration of the AI intervention. From the provider’s perspective, the clinical team felt that this intervention would potentially augment their clinical care by identifying patients at risk of requiring surgical intervention for their hydronephrosis. These opinions were aligned with the potential benefits previously outlined by the clinical team during the model development phase (11). It would also provide useful clinical decision support without adding significant time to each patient visit.

A questionnaire on the use of AI in clinical care was distributed to patients and their families after clinic visits to explore whether they would be open to consenting to use of an AI intervention and if they felt it could address their primary concerns. Out of 44 respondents, 34 (77%) prioritized knowing whether their child would require surgery as the most important, which was aligned with the primary objective of the AI intervention. Majority of respondents (68%) supported the use of AI in their care, while those who did not cited concerns that it would replace the physician-patient interaction or insufficient knowledge regarding AI itself. As a result, this questionnaire helped identify areas to educate patients and their families regarding how the AI intervention would act as a clinical adjunct to facilitate personalized and data-driven care.

## Discussion

AI integration in healthcare is growing exponentially with a diverse range of applications, from aiding diagnosis and prognosis, to supporting treatment planning and patient counseling. As more AI applications move into the clinical space, researchers have an ethical obligation to evaluate these interventions in a minimal risk environment to ensure their safety and efficacy. This is the primary motivation behind the silent trial. Here, we demonstrate the iterative changes applied to our predictive model for obstructive hydronephrosis and the resulting improvements in its accuracy and generalizability.

### Why is a silent trial warranted?

AI models trained on retrospective data alone cannot reliably function in real-world clinical settings as they are prone to dataset drift, which may include variations in how data is defined and collected, or potential changes in the standard of care if training cohorts span long periods of time (13). Use of real-time data may present additional challenges such as delays in preprocessing data or incomplete data at a given time-point. Other considerations include establishing a decision pathway and legal framework (14). A silent trial also facilitates an assessment of bias to ensure social disparities are not accentuated by the model. In this study, failure to adequately assess and account for bias may result in overtreatment of certain patient subgroups due to an inappropriately high predicted risk of obstructive hydronephrosis. We compared model performance with respect to sex, side of hydronephrosis, ultrasound machine, and patient postal code. Taken together, the silent trial enables clinicians and researchers to explore these issues in-depth without putting patients at risk of unvalidated predictions (15).

### How did the silent trial improve the applicability of our model?

While the first iteration of our model demonstrated excellent discriminative capability on a retrospective exploratory dataset (AUROC 0.90), it performed poorly on real-time data (AUROC 0.50). By validating our model in a silent trial instead of a clinical trial setting, we were able to recognize this performance drop without subjecting patients to unnecessary harm due to misclassification. Through careful consideration of dataset drift, potential sources of bias, and inclusion of other clinically relevant features, the model accuracy improved on the Silent Trial 1 dataset (AUROC 0.85) and remained robust when applied to the Silent Trial 2 dataset (AUROC 0.91). Another benefit of the silent trial is the potential to reveal discrepancies between the AI model and the current standard of care, which may highlight opportunities for quality improvement and promote hypothesis generation.

The silent trial also enables investigators to evaluate whether the proposed AI model is appropriate for real-world clinical applications. In contrast to performance evaluations which look at objective metrics, a feasibility assessment helps ensure adequate buy-in from all stakeholders. This is an essential consideration because even the most accurate AI model cannot provide meaningful clinical benefit if it is too time-consuming, difficult to use, not clinically relevant, or not endorsed by patients and physicians. In the present study, we identified nearly one-third of patients and families who were hesitant regarding the use of AI interventions to support patient care. Chew et al. found that patient concerns about AI integration were primarily attributed to a lack of trust in data privacy, patient safety, maturity of AI interventions, and risk of complete automation of their care (16). These findings underscore the need for more patient engagement prior to recruiting patients for an AI clinical trial and identifies key issues for patient education (e.g., AI will not replace their clinician, patients will still be seen by clinicians, etc.). Therefore, incorporating both patient and provider feedback into the design process can help build trust and strengthen the partnership between developers and end-users (17). Similarly, measuring patient-reported outcomes and health systems benefits in conjunction with traditional performance metrics may provide a more holistic assessment of how AI models may improve clinical practice (18). For example, a ML-based model to screen urine samples was accepted by providers because of the significant time and cost-savings without compromising care (19). Overall, the silent trial can not only refine model performance, but also facilitate a transition into clinical practice and better tailor a prospective clinical evaluation (3, 20).

### Tips to successfully implement the silent trial

Several factors outlined below are vital to the success of implementing a silent trial. The clinical team should manage sufficient patient volumes for the proposed clinical question to accrue enough patient data for the silent trial within a reasonable timeframe. The research team should have appropriate AI expertise to adequately assess the proposed model across the four themes of the silent trial. Strong partnerships between the clinical and research teams are essential and infrastructure should be established to facilitate collaboration and regular meetings between the two groups. The host institution should also be capable of storing data and the final trained model. Finally, the project should be championed by a clinical expert who can secure funding and advocate for the implementation of the AI tool into clinical practice.

### Limitations

Several limitations of this study merit discussion. The outcome of interest (obstructive hydronephrosis) was defined based on whether patients underwent surgical intervention. However, this may vary based on patient preferences, surgeon clinical judgement, and changes in clinical practice guidelines over time. Serial ultrasounds for each patient and kidney were treated as independent samples, therefore additional prognostic information from changes across serial ultrasounds may be lost. However, we felt that a rapid, point-of-care tool using single ultrasound images would be more beneficial in most clinical settings. Finally, our assessment of patient perspectives on implementation of our AI tool was based on an unvalidated questionnaire due to resource and time constraints. Future work can explore the use of validated surveys and the impact of our AI tool on patient reported outcomes.

## Conclusion

Here, we highlight our experience with the silent trial, using an AI-based classification model for hydronephrosis as an illustrative example. This phase enables stakeholders to audit and report on issues related to dataset drift, bias, feasibility, and stakeholder attitudes. These are important considerations which must be made to ensure the safety, reliability, and feasibility of AI models in real-world clinical practice. Future clinical applications of AI should make efforts to demonstrate and reflect on model changes using this process.

## Data Availability

The data is not publicly available, since all research or research-related activities that involve an external party may require, at the discretion of The Hospital for Sick Children, Toronto, Canada, a written research agreement in order to define the obligations and manage the risks.
