# Supplementary material for: The silent trial - the bridge between bench-to-bedside clinical AI applications
Source: Front Digit Health. 2022 Aug 16;4:929508. doi: 10.3389/fdgth.2022.929508 (PMC9424628; doi:10.3389/fdgth.2022.929508)
Supplement: Supplementary file 1 [file Table_1_v1.docx]

**Supplementary Material**

**Supplementary Table 1**. Post-follow-up questionnaire regarding patients’ thoughts and values of AI integration into their urologic care.

| Question | Options |
| --- | --- |
| Date of visit |  |
| Visit type | In-person  Phone call |
| I would prefer that my visits be | In-person  Video  Phone call |
| My child’s age is | 0-3 months  4-6 months  7-9 months  10-12 months  Older than 1 year |
| My child’s sex is | Male  Female |
| Today’s appointment is my child’s | First visit  Follow-up visit |
| I want to know if my child will require surgery | Rating scale from 0-100 |
| I want to know if the hydronephrosis will go away on its own | Rating scale from 0-100 |
| I would like to prevent tests that require catheters, IVs, and radiation | Rating scale from 0-100 |
| I want to decrease trips to the hospital | Rating scale from 0-100 |
| I want to know about the potential risks of infections | Rating scale from 0-100 |
| I want to know the long-term impact of this condition of my child’s kidney function | Rating scale from 0-100 |
| I want to know about the long-term outcomes of hydronephrosis | Rating scale from 0-100 |
| If an artificial intelligence (AI) tool existed to help my child's NP or Doctor make decisions about the plan of care, I would want them to use it. | Yes  No |
| Please provide any additional comments you have about using AI technology in clinical practice |  |
